# Editorial: Network-based mathematical modeling in cell and developmental biology

**DOI:** 10.3389/fcell.2024.1475005

**Published:** 2024-08-12

**Authors:** Michael L. Blinov, Susan D. Mertins

**Affiliations:** ^1^ Center for Cell Analysis and Modeling, University of Connecticut School of Medicine, Farmington, CT, United States; ^2^ BioSystems Strategies, LLC, Frederick, MD, United States

**Keywords:** cellular reaction networks, mathematical modelling, math biology, differential equations, graph theory, boolean models, whole cell modelling

The Research Topic on Network-based Mathematical Modeling in Cell and Developmental Biology raised various subjects of interest for mathematical modelers that also provide insight for future studies by bench scientists. In essence, three themes emerged and will be discussed below. First, the crucial and essential Research Topic of reproducibility of published models was addressed. Secondly, novel mathematical and computational approaches enabled a deeper understanding of biological systems. A third theme arose through the significant efforts aimed at creating whole cell models and downstream applications.

Enhancing reproducibility through rigor and transparency is a long-term goal of NIH (Collins and Tabak, 2014) and other agencies such as NSF. Wet-lab experiments can be difficult to reproduce because of variations in the conditions. Are models, essentially computational experiments, easily reproducible? Guided by FAIR principles (Findable, Accessible, Interoperable, and Reusable), Pedro Mendes examined a highly cited mathematical model that described segment polarity in *Drosophilia* published by Von Dassov et al. (2000). The unavailability of the original software forced the author to recode the model, which was a labor-intensive process that required *de novo* model implementation. The major take-home message from the report is that publication of mathematical models in a widely used standard format is essential, as only this will ensure the model is reproducible in the future.

Several novel mathematical approaches were taken by investigators to better understand cellular reaction networks. Marrone et al. described the use of nullclines, curves on a plane that are solutions to the differential equations, for analysis of systems with more than two variables. The authors followed Zhang et al. (2011) in considering pseudo-nullclines (an analog of nullclines for a system that can be decomposed into two modules) and used them to reproduce the dynamics of several well-known systems such as the embryonic cell cycle and MAPK cascade. Glazer et al. developed a new Monte Carlo Boolean Modeler (MC-Boomer) method to generate large (hundreds of thousands) collections of Boolean models whose simulations agree with observed data. A pipeline for analyzing these models and discovering novel regulatory interactions was developed and applied to a well-known model of the *Drosophila* segment polarity network (Albert and Othmer, 2003). Analysis of the models generated by MC-Boomer can be used to identify alternate hypotheses for the gene regulatory mechanism that could be then experimentally validated. Eidi et al. used stochastic modeling to investigate the spatial arrangement of cell types during stem cell division, governed by two Turing signaling patterns. Their model predicts the pattern of the differentiated cells and identifies the signaling patterns that influenced the formation of the cellular structure.

Biological networks are usually described as graphs with nodes representing biological entities (e.g., genes, proteins, functional complexes) and connecting edges representing influences on their behavior. Thus, graph-theoretical methods are under constant development, as illustrated by the two manuscripts in this Research Topic. Nam and Gunawardena introduced the linear framework—a graph-theoretic approach to analyzing biomolecular systems described by continuous time Markov processes, such as post-translational modification and gene regulation. The nodes represented individual molecular states and edges represented the probabilities of transitions between molecular states. This report described the application of linear frameworks before the steady state was reached. Specifically, the authors showed that the properties of the First Passage Time (FPT) were functions of the edge labels. The FPT defined a timescale of single-molecule kinetics, such as the enzyme’s completion time, and the approach described by the authors can be used for the analysis of real-time single-molecule data. Lecca et al. represented a biological network as a system of springs, in which the nodes constituted the masses and the edges were springs that connected these masses. Further, they defined latent geometry through the embedding of the spring network model into the metric space (Euclidean, hyperbolic, and spherical). Geometric properties of the embedded network (such as nodes clustering according to their radial coordinates) can be used for the analysis of the original biological network. The authors analyzed the transcriptome network of chronic myeloid leukemia and identified a set of candidate driver genes for network dynamics.

The third theme that emerged under the Research Topic addressed mathematical modeling beyond biochemical signaling networks. Georgouli et al. provided a review of existing multi-scale models of whole cells, starting from genome-scale models of metabolism developed in the nineties to the first whole-cell model incorporating the activity of nearly all molecules in *M. genitalium* to the recent efforts to develop the whole-cell model of *E*. *coli*. Gilbert et al. continued the theme of whole cell modeling by building a computational model of chromosome replication in a synthetic minimal bacterial cell. The authors used Langevin simulations to analyze chromosome organization. The authors noted that the polymer model of the chromosome can be used to prepare molecular dynamics models of entire Syn3A cells, validating cell states predicted by the whole-cell models. Hansen et al. discussed a topic closely related to whole-cell modeling–digital twins, multi-scale computational models of tissue and organs that work as a substitute for real human organ systems and can predict physiological events from genomic and molecular data. The authors presented opportunities and challenges for building digital twins, including parameter uncertainty, the use of artificial intelligence (AI) and machine learning (ML) methods to speed up model building, simulation and validation, and assessing the quality of predictions.

In summary, the Research Topic, Network-based Mathematical Modeling in Cell and Developmental Biology, gathered many state-of-the-art studies that will guide future directions. The melding of dry and wet laboratory studies is anticipated to advance our understanding of Systems Biology in new and exciting ways.
